# Can Generalist Predators Control *Bemisia tabaci*?

**DOI:** 10.3390/insects11110823

**Published:** 2020-11-23

**Authors:** Arash Kheirodin, Alvin M. Simmons, Jesusa C. Legaspi, Erin E. Grabarczyk, Michael D. Toews, Phillip M. Roberts, Juang-Horng Chong, William E. Snyder, Jason M. Schmidt

**Affiliations:** 1 Department of Entomology, University of Georgia, 2360 Rainwater Road, Tifton, GA 31793, USA; Arash.Kheirodin@uga.edu (A.K.); mtoews@uga.edu (M.D.T.); proberts@uga.edu (P.M.R.); 2 U.S. Vegetable Research, USDA-ARS, 2700 Savannah Highway, Charleston, SC 29414, USA; alvin.simmons@usda.gov; 3 USDA-Insect Behavior and Biocontrol Research, Gainesville, FL 32608, USA; Jesusa.legaspi@usda.gov; 4 Southeast Watershed Research, USDA-ARS, 2316 Rainwater Road, Tifton, GA 31793, USA; erin.grabarczyk@usda.gov; 5 Agriculture, Forestry and Life Sciences, Clemson University, Clemson, SC 29634, USA; juanghc@clemson.edu; 6 Department of Entomology, University of Georgia, Athens, GA 30602, USA; wesnyder@uga.edu

**Keywords:** *Bemisia tabaci*, silverleaf whitefly, sweetpotato whitefly, predatory arthropods, agroecosystems, controlled environments, open field environments, biological control, economic threshold, MEAM1

## Abstract

**Simple Summary:**

Whiteflies are major insect pests on a global scale. The use of insecticides is the primary tool for controlling them, but there are many problems relying on this strategy. However, natural enemies like predators and parasitic insects that attack whiteflies can help provide a sustainable pest management approach. This paper focuses on predators that feed on whiteflies as well as other insect pests and are called generalist predators. We provide a comprehensive view of generalist predator contributions and review the currently recognized generalist predators of whiteflies. There are many generalist predators in agricultural cropping systems that help control whiteflies. We highlight the need for conservation biological control programs through habitat management strategies and the use of selective insecticides, with an aim for more sustainable management of whiteflies in crops.

**Abstract:**

The whitefly, *Bemisia tabaci*, has developed resistance to many insecticides, renewing interest in the biological control of this global pest. Generalist predators might contribute to whitefly suppression if they commonly occur in infested fields and generally complement rather than interfere with specialized natural enemies. Here, we review literature from the last 20 years, across US cropping systems, which considers the impacts of generalist predators on *B. tabaci*. Laboratory feeding trials and molecular gut content analysis suggest that at least 30 different generalist predator species willingly and/or regularly feed on these whiteflies. Nine of these predators appear to be particularly impactful, and a higher abundance of a few of these predator species has been shown to correlate with greater *B. tabaci* predation in the field. Predator species often occupy complementary feeding niches, which would be expected to strengthen biocontrol, although intraguild predation is also common and might be disruptive. Overall, our review suggests that a bio-diverse community of generalist predators commonly attacks *B. tabaci*, with the potential to exert substantial control in the field. The key challenge will be to develop reduced-spray plans so that generalist predators, and other more specialized natural enemies, are abundant enough that their biocontrol potential is realized.

## 1. Introduction

Worldwide, the whitefly *Bemisia tabaci* Gennadius (Hemiptera: Aleyrodidae) is a major agricultural pest that causes substantial crop yield losses to cotton and vegetables [1]. This pest is problematic on crops in both field and enclosed environments, such as greenhouses. *B. tabaci* is a challenging pest to manage in part due to polyphagous feeding on greater than 1000 host plant species [2] as well as resistance to commonly effective insecticides, such as neonicotinoids [3] and pyrethroids [4]. Furthermore, many commonly used insecticides suppress parasitoid and predator populations (i.e., parasitoids and predators), which likely limits biological control options for *B. tabaci* [5,6,7]. Given the rapid resistance and declining effectiveness of pesticides targeting *B. tabaci* [8,9], understanding the role of natural enemies can improve the efficiency of management strategies to control whiteflies.

Comprehensive published reviews and books [10,11,12] all stress the significance of biological control in the management of *B. tabaci*. A recent review by Liu et al. [13] highlights the importance of *B. tabaci* parasitoids, including aphelenids, *Eretmocerus mundus* Mercet, *E. eremicus* Rose and Zolnerowich, and *Encarsia formosa* Gahan, which are widely used for successful augmentative releases. Thus, the significance of parasitoids is well documented [13]. However, the scientific community currently lacks an up to date comprehensive review regarding the role of generalist arthropod predators in the biological control of whiteflies. Over 150 species of predators around the globe have been reported to feed on *B. tabaci*, although very few have been studied in detail [13]. Understanding the role of predators is challenging because some predators may consume the same or different types of prey or pest life stages, resulting in synergistic, additive, and non-additive effects on insect pest suppression [14]. For example, if several predator species show a preference for whitefly eggs, competition and intraguild predation may result, which, in turn, could reduce the efficacy of biological control [15]. Conversely, if predator consumption of whiteflies does not overlap between species (i.e., predator complementarity) either in time or space, the efficacy of biological control may be enhanced [15,16]. Encouragingly, studies in the southwest USA provide evidence that generalist predators can contribute significantly to *B. tabaci* control [17,18,19,20]. Although past reviews identify several potential predators likely contribute significantly to *B. tabaci* biological control, except in rare examples, their contributions are not fully integrated into *B. tabaci* management.

Generalist predators contribute to pest suppression in agroecosystems by altering the behavior of or consuming major arthropod pests [21]. Several approaches have been implemented to quantify and assess the contribution of generalist predators to pest control services, such as laboratory and greenhouse feeding trials, correlative field studies, and molecular gut content analysis (MGCA) [19,22,23]. Both controlled greenhouse and open field studies are fundamental to how we understand the role of generalist predators in the biological control of *B. tabaci* [19,24,25]. In addition, a rapidly growing technique, MGCA, has been used to trace herbivore DNA in the gut of generalist predators [22,23]. MGCA has advanced researchers’ ability to screen a wide range of predators to determine their relative contribution to pest control services in agroecosystems [24]. Given the small size of the *B. tabaci* life stages, which makes observations of predation challenging, MGCA is an essential tool to unravel the range of generalist predators, contributing to its control in the agroecosystem.

In this paper, we review direct (e.g., mortality on *B. tabaci* life stages) and indirect (e.g., change in pest dispersal, colonization rate, and avoidance behavior) effects of predators on the density of *B. tabaci* in the laboratory, greenhouse, and correlative field studies conducted in the USA. Currently, the scientific community lacks a comprehensive guide that summarizes the role of predators and, in particular, generalist predators (broadly defined as those consuming multiple species or taxonomic groups) as biological control agents of *B. tabaci* in the US agricultural landscapes. Such information will help bridge the knowledge gap and bring our understanding of generalist predators up to par with parasitoid research. Therefore, the objective of our review is to identify and discuss known predator species that contribute to *B. tabaci* management in the United States. With evidence from studies over the past 20 years across the United States (since the last reviews; i.e., [4,11,12]), we review the most capable predators for integration in *B. tabaci* control at relevant ecological scales (i.e., greenhouse to landscape). Given that our review uncovers regional bias in research coverage (i.e., southwest US studies), we summarize and discuss the contribution of known generalist predator species that feed on *B. tabaci* and offer directions for future research in the southeastern US.

## 2. Materials and Methods

Our literature search was conducted in two main databases—Thompson Reuters’ ISI Web of Science and Google Scholar. We searched multiple combinations of keywords to isolate studies that focused on generalist predators and biological control of *B. tabaci*. Searches included: (*Bemisia tabaci* AND biological control), (Silverleaf whitefly AND biological control), (Bemisia tabaci*Biological control OR predation), (Sweetpotato whitefly AND biocontrol), (Sweetpotato whitefly*predation OR predator), (MEAM1*biocontrol), (*Bemisia argentifolii* AND biological control), (Bemisia argentifolii*predation OR predator), (Silverleaf whitefly*Biological control OR predation), (Bemisia tabaci*predation OR predators), (Silverleaf whitefly*predation OR predators), (whiteflies AND generalist predators), (Silverleaf whitefly predation AND United States), and (generalist predators AND Silverleaf whitefly). After finalizing the literature search, we discarded studies that were conducted outside of the United States, as the goal of this review was to directly focus on important generalist predators of *B. tabaci* present in the United States. However, we recognized that there were studies that were conducted outside of the US concerning generalist predator species that are found in the US. To review the impact of insecticides on *B. tabaci* predation, we included studies that contained data for both *B. tabaci* and predators and the impact of insecticides on *B. tabaci* predation rate and population growth under greenhouse and field scales. We also discarded all studies conducted before 2000 for two reasons: (1) several comprehensive reviews [4,11,12] and a book chapter [10] include studies conducted before 2000, and (2) our goal was to examine and report the more current information available on whitefly biological control by generalist predators (Table 1).

## 3. Experimental Evidence for Generalist Predator Importance in Whitefly Biocontrol

In this section, we review evidence of biological control by generalist predators on *B. tabaci,* first within greenhouses and laboratory studies, followed by evidence from field studies. We consider the effects of predators on *B. tabaci* populations, as well as predator preference for specific life stages, interactions among predators, and the combined effects of insecticides on both predators and *B. tabaci*. We end this section by reviewing how gut content analysis can further shape our understanding of the role that generalist predators might play in whitefly suppression.

### 3.1. Direct Predation of Bemisia tabaci by Generalist Predators

Laboratory and greenhouse studies shed light on the potential natural enemies of *B. tabaci*. Such fine-scale studies can give an initial indication of a generalist predator’s (1) voracity for and preference for whitefly life stages and (2) potential to engage in intraguild predation. However, this information includes the substantial caveat that species interactions might be substantially different in simplified laboratory arenas, compared to the much more complex environment of an open agricultural field.

Broad diversity of generalist predator species has been examined for *B. tabaci* in simplified laboratory and greenhouse arenas. These include six Coleoptera (beetle species), 11 Hemiptera (true bug), two Neuroptera (lacewing), one Diptera (fly), eight spider species, and two mite species (Table 2). The bulk of information on predators comes from laboratory and greenhouse studies focused on documenting predation in very controlled environments (Table 1).

Greenhouse experiments demonstrate that various predator species contribute to direct control of *B. tabaci* in a variety of crops. For example, on tomato plants, *Dicyphus hesperus* Knight (Hemiptera: Miridae) led to a decline in the density of *B. tabaci* eggs and nymphs and up to 60% suppression of nymphal stages [32]. Similarly, *Serangium parcesetosum* significantly suppressed the population growth of *B. tabaci* on poinsettia (*Euphorbia pulcherrima*) plants within six weeks of their release [25]. Populations of *Amblyseius tamatavensis* Blommers (Acari: Phytoseiidae) colonize nightshade plants (*Solanum americanum*) in southern Florida, and in a laboratory experiment, fed on *B. tabaci,* suggesting that this species can be a useful predator of *B. tabaci* in the southeast USA [30].

### 3.2. Placing Generalist Predators in a Community-Ecology Context

Generalist predators often forage within diverse communities of other natural enemy species, including both other generalists and more specialized predators and parasitoids. Sometimes, generalist predators occupy different feeding niches than other sympatric natural enemy species, attacking different pest species or stages, foraging in different places or times or using different means of attack. That is, different species complement one another, which, in turn, can lead to broader-based overall attacks on the pest when several natural enemy species co-occur. Other times, however, generalists heavily attack other natural enemies, and this intraguild predation can disrupt the overall strength of biocontrol. Intraguild predation can be particularly harmful when generalist predators with relatively broad feeding niches focus their attacks on the specialized natural enemy that would otherwise be the key biocontrol agent, suppressing a particular pest species [60,61]. Finally, generalist predators often attack a broad diversity of prey species that are not pests or natural enemies or will feed on plants or consume plant pollen or nectar. Sometimes, feeding on these “alternative” foods can help build numbers of generalist predators that then are available to attack pests, strengthening biological control. But in other cases, the alternative foods only draw attacks of generalist predators away from attacks on the target pest, with this distraction weakening overall biocontrol by generalists. Our literature review reveals evidence for both complementarity and interference among *B. tabaci* natural enemies and for helpful versus harmful effects of alternative foods, and we next describe each of these ecological complications in turn.

#### 3.2.1. Complementarity among Generalist Predators of *Bemisia tabaci*

**Figure 1:**
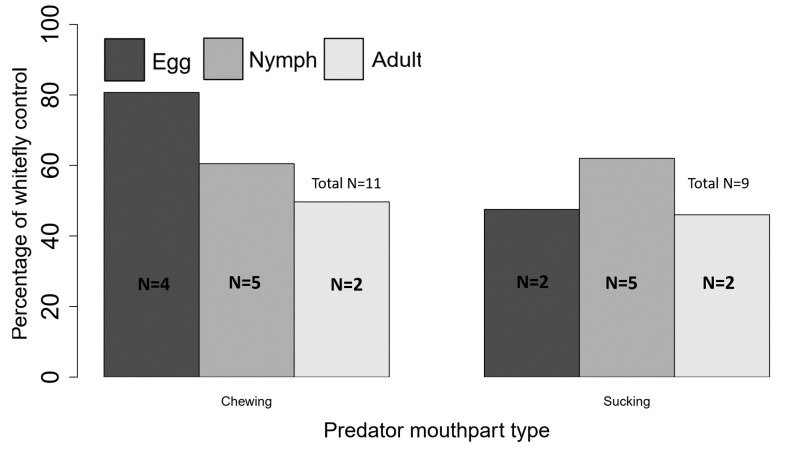
Contribution of generalist predators on stage-specific *Bemisia tabaci* control with chewing (11) and sucking mouthparts (9). The total number of studies used for calculation of the percentage of whitefly life stages control is indicated by “N” for each life stage and each mouthpart type. The percentage control was calculated by averaging the predation rate or percentage of whitefly population growth control across all studies.

Complementarity resulting from different natural enemy species attacking different whitefly life stages is perhaps best documented in the literature. For example, recent laboratory work reveals that *D. catalinae*, *Collops vittatus* (Say, Coleoptera: Melyridae), *Hippodamia convergens* Guérin-Méneville (Coleoptera: Coccinellidae), and *D. pallidus* differ in their preference for consuming *B. tabaci* eggs versus small (1–3rd instars) or large nymphs (4th instar) [23,35,38]. These differences among species appear to be driven by differences in mouthpart morphology. Two chewing predators, *C. vittatus* and *H. convergens*, preferentially attack *B. tabaci* eggs but also fed on nymphs [38]. In contrast, two piercing-sucking predators, *Orius tristicolor* (White, 1879) (Hemiptera: Anthocoridae) and Geocoris punctipes (Say, 1832) (Hemiptera: Geocoridae), preferentially attack adult *B. tabaci*, while *Lygus hesperus* (Knight, 1917) (Hemiptera: Miridae) more commonly attacks nymphs [38]. This suggests that common whitefly predators in cotton fields may feed on all life stages of *B. tabaci,* and synergism appears possible when chewing and sucking predators are found together, which would enhance the suppression of *B. tabaci* (Figure 1). Combined, predator foraging traits for different *B. tabaci* life stages would result in complementarity, and hence, enhancing predator diversity could be key to improving *B. tabaci* biological control in the agroecosystems.

For example, coccinellids, including *Delphastus catalinae* (Horn), *D. pallidus* LeConte, and *Nephaspis oculatus* (Blatchley), have shown a type two functional response towards *B. tabaci* eggs; predators have consumed a lower proportion of eggs as the number of offered eggs increased [33,35]. Conversely, Rincon et al. [50] observed a type three response better fit the number of *B. tabaci* consumed by *D. catalinae* within tomato plants. Interestingly, despite uniform distributions of *B. tabaci* across the tomato plants, *D. catalinae* has primarily foraged on lower and middle nodal leaves that might have led to microsites of predator-free space for whitefly on higher plant nodes [50].

#### 3.2.2. Intraguild Predation among Generalist Predators of *Bemisia tabaci*

Generalist predators pose trade-offs and are known for their negative effects via competition and intraguild predation, which diminish the efficacy of biological control [21]. The occurrence of intraguild predation between predators (*G. punctipes, O. insidiosus,* and *H. convergens*) and the parasitoid, *Eretmocerus sp. nr. emiratus* (Hymenoptera: Aphelinidae) feeding on *B. tabaci* 4th nymphal stage, has been evaluated under laboratory conditions [41]. In a choice trial, all predators are significantly fed on parasitized nymph and pupa (higher preference) at a higher rate relative to unparasitized *B. tabaci* 4th instar nymphs. This suggests a high probability of intraguild predation between *B. tabaci* predators and parasitoids. Under the no-choice condition, *G. punctipes* and *O. insidiosus* have consumed all types of given prey without a significant preference. *H. convergens*, however, has a more complex response, where it consumes significantly more parasitized pupa and fewer *B. tabaci* nymphs. Some predators show flexible responses and may not discriminate between parasitized and non-parasitized prey and, as a result, may outcompete more specialized whitefly natural enemies. Similarly, Zang and Liu. [40] found evidence of intraguild predation in the greenhouse, where they found that *D. catalinae* fed on parasitized *B. tabaci* nymphs on cabbage plants and that *Encarsia sophia* (Girault and Dodd) (Hymenoptera: Aphelinidae) parasitized 1.2-fold fewer *B. tabaci* nymphs when *D. catalinae* was present. Overall, *B. tabaci* suppression was higher when *D. catalinae* was present (e.g., it preyed on to 90% of individuals by the last sampling date), regardless of its potential effect as an intraguild predator, suggesting while intraguild predation does occur, in this case, the benefits outweigh the costs. Although preliminary, these results suggest a high probability for intraguild predation between *B. tabaci* predators and parasitoids under field conditions, but the benefits to biological control appear to outweigh the trade-offs of more complex interactions.

### 3.3. Non-Trophic Impacts of Generalist Predators on Bemisia tabaci

The presence of natural enemies can alter *B. tabaci* movement within and between plants, which may indirectly impact the colonization of, and damage by, whiteflies. Of particular interest is when the presence of natural enemies influences the dispersal of *B. tabaci* from a cash crop to a trap crop. In a greenhouse study, the colonization rate and density of *B. tabaci* are significantly higher on cucumber (trap crop) relative to poinsettia (cash crop) in the presence of *Amblyseius swirskii* Athias-Henriot (Mesostigmata: Phytoseiidae), *D. catalinae*, and the parasitoid *E. formosa* [48]. Furthermore, in a laboratory leaf disc experiment, *B. tabaci* has delayed settling on cucumber leaves in the presence of *D. catalinae*, suggesting an avoidance behavior when *D. catalinae* is present [47], and within an entire plant, *B. tabaci* adults move towards the top of cucumber plants in response to the presence of *D. catalinae* [47]. Thus, the presence of *D. catalinae* alters *B. tabaci* host choice and dispersal from cash to trap crops but also small-scale movement within a single plant [47,48]. Given only a few studies to draw conclusions, initial results suggest predators likely impact *B. tabaci* population growth directly through predation and indirectly via non-consumptive effects, such as dispersal.

## 4. Within Field Studies of Generalist Predators of *Bemisia tabaci*

Although greenhouse and laboratory studies have greatly contributed to our understanding of the direct and indirect effects of predators on *B. tabaci*, larger-scale field and landscape studies have the potential to test these interactions under realistic environmental conditions. In this section, we review work at field and landscape levels and show that additional research is needed to better integrate generalist predators into sustainable control of *B. tabaci* in the United States.

### 4.1. Life-Table Analysis of Generalist Predator Effects on Bemisia tabaci

Field studies that include life-table observations highlight the importance and contribution of several generalist predators to specific stages of *B. tabaci*. In cotton, fourth instar nymph mortality is primarily due to consumption by predators with piercing-sucking mouthparts and predators with chewing mouthparts [36]. Egg and 1st instar predation is correlated with the density of *O. tristicolor* (Hemiptera: Anthocoridae), *C. vittatus*, *Chrysoperla carnea* s.l. Stephens (Neuroptera: Chrysopidae), and most strongly with *G. punctipes* density [36]. Additionally, third and fourth instar nymphal mortality is linked to the same above species along with *L. hesperus* and *Geocoris pallens* Stal (Hemiptera: Geocoridae) [44,45,46]. Consistently, in a long-term life-table study spanning 14 years, the highest mortality attributed to generalist predators is on fourth instar *B. tabaci* nymphs, followed by eggs and other nymphal stages [20]. Furthermore, initial results from incorporating natural enemies into the current economic threshold for *B. tabaci* also suggest life stage-specific contributions in whitefly control [19]. In cotton, the ratio of *C. carnea* larvae/*B. tabaci*, *Drapetis. nr. divergens* (Diptera: Empididae) and *Misumenops celer* (Hentz) (Araneae: Thomisidae)/*B. tabaci* has reduced the proportion of leaves, surpassing the economic threshold. Further, low density and *B. tabaci* infestation rate are most consistently associated with the ratio of *D. nr. divergens*, *M. celer,* and *G. pallens*/*B. tabaci* in cotton fields. The ratio of six predator species/*B. tabaci* is significantly associated with motility of various *B. tabaci* life stages, providing further support for potential synergism (e.g., complementary predation) among chewing and sucking predators. Consequently, results from the southwest provide substantial support for several predator species in cotton fields that readily feed on various life stages of *B. tabaci* and contribute significantly to its population suppression (Table 3).

### 4.2. Molecular Gut Content Analysis to Determine Predators of Bemisia tabaci

The density of herbivores in agricultural fields often depends on the density of various natural enemies. However, the extent to which natural enemies contribute to pest control services, especially for small insects, such as whiteflies, is often difficult to estimate with traditional laboratory, greenhouse, or field techniques. Laboratory and greenhouse studies often present unrealistic environments that may under or overestimate the contribution of predators to pest control services for fields [62]. In agricultural fields or landscapes, identifying the specific predator that contributes to control is not always clear when correlating diversity indices with pest populations. Moreover, several predator species, including *A. swirskii* [31,49] and *D. catalinae* [28,35], which significantly suppress whitefly population growth under greenhouse conditions, are not abundant in the field [17,46,47], and therefore, their natural field contribution to biological control is considered minimal. Although predator exclusion cage studies in the field can reveal important information regarding the overall impact of predators on pest population [63], cage studies do not identify the predator species responsible for the observed control. MGCA, molecular gut content analysis, is a technique that enables tracking of predator diets and helps sort predators into those actually feeding on pests in agricultural fields and landscapes [21,64]. MGCA can detect predators of whitefly under field conditions [17] and can be the foundation for the development of a biologically informed threshold for *B. tabaci* [19]. Below we review recent research that employs MGCA to identify predators that commonly feed on *B. tabaci*. Laboratory research with *Orius insidiosus* Say and *G. punctipes* when provided adult *B. tabaci* suggest that the interpretation of feeding impact based on MGCA could be skewed based on the half-life detection within a specific predator to prey combination [65]. Yet, contrary to their hypothesis, the data did not support longer half-lives of gut analyses in hemipterans versus other predators.

A handful of MGCA studies in the USA have confirmed predation on all life stages of *B. tabaci* by multiple predators [17,40,41]. Hagler and Naranjo. [51] reported predation on whitefly eggs and adult females by *H. convergens*. Using a similar approach, Hagler and Naranjo [18] tracked *B. tabaci* eggs and adult females’ protein in the gut of several abundant predator species in Arizona cotton fields. In addition to being the most abundant in cotton fields, these species were frequently observed feeding on *B. tabaci* (Table 3). Similarly, Hagler and Blackmer [17] found a high percentage of various predators with *B. tabaci* DNA in their gut from cotton fields (Table 3). Contrary to these findings, predation during the early, mid, and late season in Georgia cotton and predation on *B. tabaci* by species within two spider families, including Gnaphosidae and Salticidae, was low [59]. The difference between studies may be due to overall higher densities of whitefly in Arizona cotton fields relative to Georgia. Combined, these initial studies confirm region-specific predation on whiteflies and highlight the need to understand responses across a broader spatial scale, other systems, and understand field conditions influencing predation levels (Table 3).

## 5. Conserving Generalist Predators to Strengthen Biocontrol

**Figure 2:**
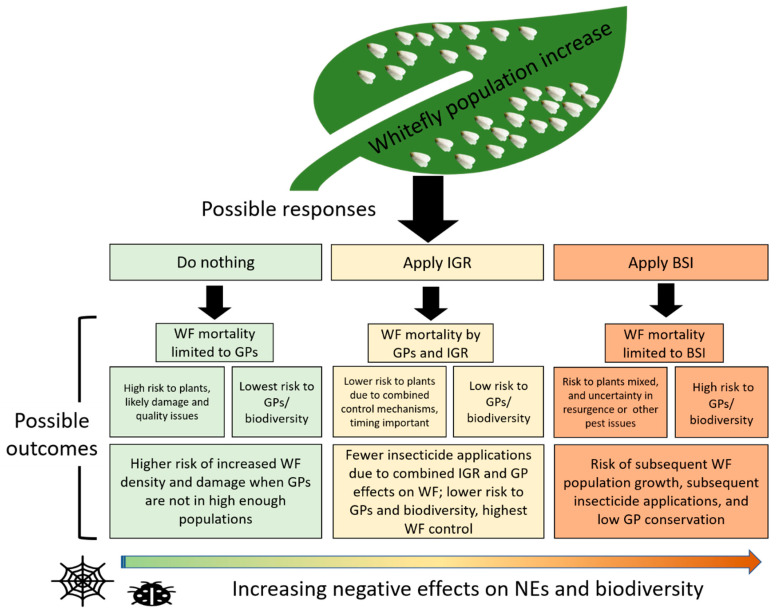
Several studies conducted in the southwest US cotton fields have highlighted the significant adverse effect of insecticides on *Bemisia tabaci* control and associated predators [27,42,43,44,45,46]. Here, we summarize these effects using a conceptual chart to represent the currently predicted impacts of three contrasting insecticide regimes on generalist predator conservation in cotton fields and its effect on *B. tabaci* control in the US agroecosystem. The abbreviations are as follows: WF: whitefly, *B. tabaci*; GP: generalist predators; IGP: intraguild predation; IGR: insect growth regulator; BSI: broad-spectrum insecticide.

The International Organization for Biological Control and suppliers of biological control agents (including some generalist predator species) for augmentative releases in fields and greenhouses maintain databases on the compatibility of pesticides with biological control agents (such as [66,67,68,69]). These databases are based mainly on laboratory bioassays, but they represent valuable resources to growers who are interested in integrating biological and chemical management. They, however, do not specifically indicate the effects of pesticide applications on whitefly biological control in the fields or greenhouses. Nine studies have documented the effects of pesticides on predator-*B. tabaci* interactions in agroecosystems in the US (Table 1). These studies have demonstrated that the contributions of generalist predators to whitefly population suppression in agroecosystems could be negatively affected by insecticide applications. However, these studies have also indicated the potential for integrating chemical and biological control through careful selection of insecticides and planning of application timing. A conceptual framework for the overall effects of different chemical management strategies in relation to whitefly biological control and pesticide resistance management is presented in Figure 2, where an increasing reliance on broad-spectrum insecticides will likely lead to a greater reduction of generalist abundance and efficacy and risk of pesticide resistance development in *B. tabaci* populations. We recognize that additional research is needed to understand the broader effects of pesticide application on a greater number of generalist predator species and to identify the trade-offs between chemical and biological control.

We emphasize the importance of relying on economic thresholds when initiating chemical management strategies. However, there may be benefits in modifying economical thresholds (developed largely for pesticide application) to account for the potential contribution of biological control in the management of *B. tabaci*. Naranjo et al. [44] found that allowing higher populations of whiteflies (i.e., relaxing economic threshold) helped maintain predator abundances and predation efficacies similar to the untreated control for an extended duration and resulted in a reduction in whitefly abundance on cotton.

One of the most influential ways chemical management may impact the efficacy of whitefly biological control with generalist predators is through the selection of insecticides. It is generally recognized that broad-spectrum insecticides, such as carbamates, organophosphates, and pyrethroids, can have devastating consequences for predators [68]. Applications of a mixture of fenpropathrin (a pyrethoid) and acephate (an organophosphate) significantly reduce the abundance of several important *B. tabaci* predators (e.g., *G. punctipes*, *G. pallens*, *O. tristicolor*, *Drapetis* sp., and *H. convergens*) and predation, reducing the effectiveness of biological control in cotton [44]. Asiimwe et al. [27] also reported a significant reduction in natural enemy density in plots that had been treated with acephate compared to untreated control. Even pesticides that are generally considered compatible or organic may negatively impact natural enemy abundance and activities. For example, both M-Pede (potassium salts of fatty acids) and PyGanic (pyrethrins) significantly suppress *B. tabaci* populations relative to the untreated control in squash, but the 1-day-old residue of these bioinsecticides also suppresses the density of introduced D. catalinae and may diminish biological control [29].

There appears to be a good support for the benefits of insect growth regulators (IGR) in maintaining predator abundance in field crops [18,19,44,45]. IGRs can impact the development of immature predators, which results in a lower predator density in IGR-treated plots relative to untreated control [18]. Compared to the impacts of broad-spectrum insecticides, however, IGRs have the benefits of allowing the predator populations to recover faster after the cessation of application. Naranjo and Ellsworth [43] compiled extensive long-term data on *B. tabaci* life-tables to assess the effect of three pest management strategies (no insecticide, IGR, and broad-spectrum insecticides) on *B. tabaci* control in Arizona cotton fields. Overall, they found predator populations recovered rapidly following buprofezin and pyriproxyfen (both IGRs) treatments and contributed to *B. tabaci* control, while predator populations were significantly reduced after the use of broad-spectrum insecticides (fenpropathrin, acephate, endosulfan, and oxamyl). Moreover, the effect of commonly used insecticides (e.g., endosulfan and fenpropathrin) became ineffective, requiring up to four additional insecticide applications to maintain whitefly density below the economic threshold [43]. Conversely, the use of IGR (buprofezin and pyriproxyfen) consistently maintained whitefly populations below the economic threshold. The authors provided two mutually non-exclusive explanations: (1) high efficiency of IGR products against *B. tabaci*, and (2) the conservation of natural enemies in cotton fields, which resulted in higher levels of whitefly control (Figure 2). The use of IGR for whitefly control is, therefore, consistent with conservation biological control practices of using specific insecticides.

The negative impacts of pesticides on predators can be moderated with carefully planned treatments of applications. Razze et al. [29] demonstrated that while *D. catalinae* abundance was low on squash plants with the 1-day-old residue of M-Pede and PyGanic, the number of beetles found on plants with the 5-day-old residue of the insecticides were similar to those on the untreated plants. Each insecticide has its effective residual longevity, which is influenced by the target insects (pests or natural enemies), various environmental factors, and the inherent chemical properties of the active and inert ingredients. However, the interactions among pesticide degradation, predator tolerance for pesticide residue, and the level of whitefly suppression are poorly understood at this time.

## 6. Current Synthesis of Predator Roles in the US Whitefly Biocontrol

### 6.1. Open Field Agroecosystems

Our review provides extensive information on predators of *B. tabaci* in the US (Table 2 and Table 3) and highlights the importance of predators and their relative contribution to *B. tabaci* control. Importantly, we show that generalist predators suppress *B. tabaci* in agricultural fields and identify the potential for predator complementarity, which may boost current biological control. However, there are significant limitations in our current state of knowledge regarding the effectiveness of generalist predators in the US agroecosystems. To date, all studies reported at the field scale [19,27,42,43,44,45,46] are in cotton fields and mostly from the southwestern region of the US. Currently, we have no information regarding the community of predators in other cropping systems and their contributions to *B. tabaci* control. Therefore, the conclusions of this review are primarily applicable to *B. tabaci* control in cotton fields and greenhouses. Although over 30 species of generalist predators contribute significantly to biological control (Table 2) in the US, several are capable of suppressing *B. tabaci* below economic thresholds in the greenhouse or in cotton cropping systems [19,28,30]. We have identified seven species that appear to be effective predators (Table 3). Among the species identified, predation varies across *B. tabaci* life stages. For example, coleopterans prefer *B. tabaci* eggs (*C. vittatus* and *D. catalinae*, very successful in greenhouses). In contrast, hemipterans (G. *pallens* and *O. tristicolor*) prefer B. tabaci nymphs and adults, and *D. nr. divergens* consume both adults and eggs (Table 2, Figure 1). Accordingly, enhancing combinations of these predators at critical times during the season has the potential to control all *B. tabaci* life stages, a finding that is consistent with creating the “right kind of biodiversity” for pest management [70,71]. Additional research may incorporate the contributions of these natural enemies into current economic thresholds for *B. tabaci*. Some of the best work to date proposes predator-pest ratios for scouting fields to make management decisions [19], and this work needs to be conducted in other regions and crops. Furthermore, management that incorporates knowledge of predator-pest interactions has the potential to lower dependence on insecticides and improve the sustainability of management, targeting *B. tabaci* in the US agroecosystems. Alternatively, selective insecticide applications may decrease the loss of natural enemy populations, which may result in a more sustainable control method and higher suppression of *B. tabaci* (Figure 2). Several predators contribute to *B. tabaci* control, and conserving these predators could result in the sustainable management of *B. tabaci* in the US agroecosystem (Table 3, Figure 2).

### 6.2. Controlled Environment Agroecosystems

Growing crops in controlled environments pose both benefits and challenges regarding managing crop pests. On the one hand, the enclosure provides some protection from pests. On the other hand, once pests get in, they tend to be protected from feral natural enemies. Because crops can be grown year-round in controlled environments, not having a crop-free cycle may be conducive to the build-up of whiteflies. Unfortunately, *B. tabaci* is problematic in greenhouses across the US, especially in vegetable and ornamental crops. Fortunately, the enclosure environment keeps released predators from leaving the cropping system. Several species of natural enemies are commercially sold to help manage whiteflies in controlled environments, including predators, such as predatory mites, *Orius* spp., and *D. catalinae*, and methods are under study to monitor biocontrol agents [56,57,58].

### 6.3. Impact of Environmental Stress

Because there are different climates and environments across the US, one would expect some predators may be more effective against whiteflies in some environments than in others. Only a few reports document the impact of the environment on the populations of whitefly predators in the US. Several laboratory studies have been conducted, which define the temperature and humidity ranges for *D. catalinae* [53,55,56,58]. This predator can survive the similar temperature and humidity conditions where *B. tabaci* survives. Low humidity (5.04 mb) compared with higher humidity (up to 30.25 mb) is found to have an adverse impact on the populations of *D. catalinae*. In the field environment, this predator is demonstrated to survive the mild winter in the same field environment where the host survives year-round [53].

## 7. Future Research Opportunities

### 7.1. Expanding the Geographical Range and Scale

Currently, our knowledge regarding whitefly predation is limited to field studies in the southwest and primarily laboratory and greenhouse trials in the southeastern United States. The majority of research on whiteflies and predators is derived from southwestern cotton systems. Because the distribution of whitefly is expanding into the southeastern US, solutions for this region are of critical importance. Research has focused primarily on laboratory and greenhouse systems (Table 1 and Table 2); therefore, there is a need for research to be conducted at the commercial field (i.e., 16–80 ha) and landscape scales (i.e., 1–5 km) to better understand and incorporate the effect of predators in *B. tabaci* management.

Investigations that cover broad spatial scales are required to understand how agricultural landscape complexity around crop fields affect the recruitment of *B. tabaci* natural enemies into fields and associated rates of control. Landscape simplification may negatively affect insect pest biological control due to a reduction in the richness and abundance of natural enemies in agricultural landscapes [72]. Agricultural landscape complexity affects herbivore density in crop fields in direct (e.g., resource concentration hypothesis) and indirect ways (e.g., enemies hypothesis [73,74,75]). To our knowledge, no US studies have evaluated the effect of landscape complexity on *B. tabaci* abundance and its predation. Further, while several studies have reported the potential predators of *B. tabaci* [17,19,20], the effect of predator richness and diversity in response to landscape structure on *B. tabaci* population dynamics has not been studied in the US agroecosystems. To build area-wide pest management strategies for *B. tabaci*, research is needed not only in cotton fields but also in vegetables. This is particularly important because generalist predator communities are likely unique to each cropping system, and in the southeast, crop systems overlap in space and time, which provides host sources for *B. tabaci* and generalist predator populations.

Presently, there is a dearth of information available on predation levels on whiteflies at field or landscape scales. Molecular gut content analysis (MGCA) is an effective method to determine the links between trophic levels in agricultural fields [22,76]. Given the potential of MGCA to unravel natural enemy interactions with pests [23,24,77,78,79], it is surprising that only a few studies have determined key predators of *B. tabaci* in the US agroecosystems [17,59]. Current understanding of natural enemy-*B. tabaci* trophic interactions will benefit from studies conducted with a gradient of agricultural land-use practices with a broad spatial extent to model not only the movement of but also trophic interactions between cropping systems.

### 7.2. Integrating Generalist Predators into Economic Thresholds

Few studies have successfully incorporated the effect of natural enemies into the economic threshold for herbivorous insect pests [80,81]. Although predator-pest ratios have been incorporated into *B. tabaci* management strategies, to some degree in the southwest [19], the same predator-pest ratios are not applicable to other regions. Predator communities differ in the southwest and southeast. The proposed predator-pest ratios may or may not fit the conditions for the southeast. For example, while some of the dominant predators in the southwest, including Coccinellidae, are abundant in the southeast, others, such as *Orius,* are primarily a different species, and Thomisidae and Empididae have been observed less frequently in recent southeast studies [82]. Therefore, evaluating and optimizing ratios of regional predator communities are essential next steps to build natural enemy focused thresholds. This approach is holistic and could result in lower insecticide applications; however, current thresholds do not incorporate the roles of plant and soil health components. For instance, how the levels of fertilizer or cover crop strategy components impact plant resistance to insect damage, and subsequently, the levels of yield loss due to that damage [83]. Moreover, the calculation of predator-prey ratios might be time-consuming, and many growers may lean towards the preventative insecticide applications. An exciting development made possible by the rise in smartphone use is integrating natural enemy and pest data within a user-friendly application (e.g., Agriculture and Agri-Food Canada, [84]), making calculations easier for farmers and helping guide insecticide applications. In closing, our review provides many avenues for future research in the United States to improve biological control of *B. tabaci* in agroecosystems.

## 8. Conclusions

With the great diversity of predators observed for whitefly control and success of integration, this system is ripe for continued research to optimize integrated pest management approaches that better harvest these biocontrol services. Advances in the southwest [19] are excellent blueprints for incorporation into the southeast environments. The southeast presents additional challenges as the climate is subtropical to tropical with very mild winters and many non-cropping habitats that may act as hosts. In addition, the agricultural landscape mosaic over the season, due to mild climatic conditions, is dynamic with upwards of three cropping seasons. While there are good systems in place for using both parasitoids and predators in controlled environments to regulate *B. tabaci*, from our review, working at field scales and across landscapes will help move biocontrol efforts forward.

## Figures and Tables

**Table 1:** Summary of studies focused on predators and whitefly (*Bemisia tabaci*) interactions. Several concepts explaining the direct and indirect impact of predators on *B. tabaci* population control in the laboratory, greenhouse, and US agroecosystem were reviewed from the studies conducted in the past 20 years. Further, factors affecting the direct effect of predators on *B. tabaci* control were reviewed (e.g., insecticides). We divided the studies into conceptual frameworks and recorded the number of studies in each concept category with associated references. The “#” indicates the number of studies tested the predator effect under each spatial scape.

Concept Studied	Lab#	Greenhouse#	Field#	Total#	References
Direct effect of predators on whitefly population growth and control	2	5	6	13	[19,20,25,26,27,28,29,30,31,32,33,34,35,36]
Predator prey life stage preference and host plant preference	5	0	0	5	[34,35,37,38,39]
Predator gut content analysis	1	0	5	6	[17,18,37,40,41]
Intraguild predation among predators and parasitoids	2	0	0	2	[40,41]
Insecticide effects on *B. tabaci* predation	0	1	8	9	[18,19,27,29,42,43,44,45,46]
Indirect effects of predators on *B. tabaci* dispersal	0	2	0	2	[47,48]
Thresholds and predator-prey ratios testing	0	0	2	2	[19,44]
Banker plant effect on *B. tabaci* biocontrol	0	2	0	2	[28,49]
Predator movement pattern from release site	0	1	1	2	[50,51]
Environmental stress effect on predator	3	0	1	4	[52,53,54,55]
Compatibility with whitefly sticky traps	1	2	0	3	[56,57,58]

**Table 2:** Summary of *Bemisia tabaci* generalist predators in the United States, along with information regarding the spatial scale of the study (e.g., field, laboratory or greenhouse), the levels of reported contribution to *B. tabaci* control under the various setting.

**Order: Family**	**Species**	**Crops**		**Spatial Scale**		**Prey Stage Preference**	**Preferred Crop**	**Control and/or Predation Rates**	**References**
Coleoptera*:*	*Delphastus catalinae*	Cotton, collard, tomato, hibiscus, and squash		Laboratory, greenhouse and field		Eggs	Cotton	98.6% in petri-dish, 75% population growth reduction in the greenhouse	[23,25,30,35,52]
Coccinellidae	*Hippodamia convergens*	Cotton and cantaloupe		Laboratory and field		Eggs, parasitized nymphs	Not studied	45.5% nymphal mortality petri-dish, 50% of individuals positive for *B. tabaci* DNA	[17,38,43]
	*Delphastus pallidus*	fiscus hedge, cotton		Laboratory		Eggs & early nymphs	Not studied	68.0% and 55.1% eggs and nymph mortality on leaf disc, respectively	[35,39]
	*Serangium parcesetosum*	Poinsettia		Greenhouse		Not studied	Not studied	Up to 60% *B. tabaci* mortality with four individuals per plant	[28]
	*Nephaspis oculatus*	Collards, soybean and tomato		Laboratory		Not studied	*Collards*	72.55% average egg predation on leaf discs at range of egg densities in 24 h	[34]
Melyridae	*Collops vittatus*	Cotton		Laboratory and field		Eggs & adults	Not studied	86% positive for *B. tabaci* DNA in cotton fields.13.1 eggs consumed per hour. Reduce *B. tabaci* immatures	[17,19,38]
Hemiptera:	*Geocoris punctipes*	Cotton		Laboratory and field		Adult and parasitized nymphs	Not studied	36% nymphal predation in petri dish, predation on 4th instar nymph considered *B. tabaci* key mortality factor in cotton fields	[29,36,38,43,46]
Geocoridae	*Geocoris pallens*	Cotton		Field		Adult	Not studied	Considered one of the key predators of *B. tabaci* with predator-prey ratios of 0.75 *G. pallens* per 100 sweeps to one large *B. tabaci* nymph	[17,19,29,36]
Anthocoridae	*Orius tristicolor*	Cotton		Laboratory and field		Adult and nymphal stages	Not studied	In the laboratory, adults consumed up to 2.1 *B. tabaci* adults per hour. In fields, considered key predators of *B. tabaci*	[18,19,29,38]
**Order: Family**	**Species**	**Crops**		**Spatial Scale**		**Prey stage Preference**	**Preferred Crop**	**Control and/or Predation Rates**	**References**
Miridae	*Spanagonicus albofasciatus*	Cotton		Field		Not studied	Not studied	A range of 30–50% tested positive for *B. tabaci* eggs or adult females’ antigen	[18]
	*Lygus hesperus*	Cotton		Laboratory and field		nymphs	Not studied	Adults consumed up to 2.4 *B. tabaci* nymphs per hour. Also, it is a cotton pest. Its density was correlated with predation on *B. tabaci* life stages in cotton fields	[19,36,38]
	*Pseudatomocelis seriatus*	Cotton		Field		Not studied	Not studied	A range of 30–50% *P. seriatus* individuals fed on *B. tabaci* eggs or adult females	[18]
	*Dicyphus hesperus*	Tomato		Greenhouse		Not studied	Not studied	Up to 70% and 64% *B. tabaci* egg and nymph population control over six weeks	[33]
	*Rhinacloa forticornis*	Cotton		Field		Not studied	Not studied	Not significant or minimal levels of control in cotton fields	[19,24,36]
Reduviidae	*Zelus renardii*	Cotton		Field		Not studied	Not studied	On average, 49.3 % of individuals *Z. renardii* had *B. tabaci* DNA in their gut	[17]
	*Sinea confuse*	Cotton		Field		Not studied.	Not studied	On average, 15.3% of individuals had *B. tabaci* DNA in their gut	[17]
Nabidae	*Nabis alternatus*	Cotton		Field		Not studied.	Not studied	On average, 32.4% of individuals had *B. tabaci* DNA in their gut	[17]
Diptera: Empididae	*Drapetis nr. divergens*	Cotton		Field		Adults and eggs	Not studied	The predator-*B. tabaci* ratio of 8 or 44 *D.* nr *divergens* per 100 sweeps to one adult or one large *B. tabaci* nymph. 32.4% of individuals positives for *B. tabaci* DNA	[18,19,37]
Neuroptera: Chrysopidae	*Chrysoperla carnea*	Cotton		Field		Not studied	Not studied	49% positive for *B. tabaci* DNA. Significant negative effect on *B. tabaci* immature stages in cotton fields	[17,19,36]
Acari: Phytoseiidae	*Amblyseius swirskii*	Green bean and pepper		Greenhouse		Not studied	Pepper	Significant suppression of the *B. tabaci* population on green bean plants by *A. swirskii* in the greenhouse	[32,50,51]
	*Amblyseius tamataven*	black nightshade		Laboratory and field		Not studied	Not studied	Can complete development on *B. tabaci*, suggesting suitability	[31]
**Order: Family**	**Species**	**Crops**	**Spatial Scale**		**Prey stage Preference**	**Preferred Crop**	**Control and/or Predation Rates**		**References**
Araneae Thomisidae	*Misumenops celer*	Cotton	Field		Not studied	Not studied	31% and 32.5% individuals tested positive for *B. tabaci* antigen and DNA, respectively. Significant reduction in *B. tabaci* density in cotton fields with high *M. celer* density		[17,18,19,24]
Dictynidae	*Dictyna reticulata*	Cotton	Field		Not studied	Not studied	On average, 39.5% of individuals had *B. tabaci* DNA in their gut		[17]
Clubionidae	*Clubiona* spp.	Cotton	Field		Not studied	Not studied	31.6% positive for *B. tabaci* DNA		[17]
Salticidae	No species identity	Cotton	Field		Not studied	Not studied	8% positive for *B. tabaci* DNA		[17,59]
Lycosidae	*Hogna* spp.	Cotton	Field		Not studied	Not studied	22.2% positive for *B. tabaci* DNA		[17]
Araneidae	No species identity	Cotton	Field		Not studied	Not studied	25% positive for *B. tabaci* DNA		[17]
Miturgidae	*Cheiracanthium inclusum*	Cotton	Field		Not studied	Not studied	71.4% positive for *B. tabaci* DNA		[17]
Corinnidae	*Trachelas* spp.	Cotton	Field		Not studied	Not studied	An average of 33.3% of individuals was positive for *B. tabaci* DNA. But the species had a low density		[17]
Gnaphosidae	No species identity	Cotton	Field		Not studied	Not studied	An average of 50% of individuals was positive for *B. tabaci* DNA. But the species had a low density		[17]

Notes: The proportion positives reviewed in this table may contain the secondary predation values through intraguild predation, which has not been specified in these studies.

**Table 3:** Summary of the most abundant and significant predators of *Bemisia tabaci* in fields and greenhouse conditions.

Predator ^1^	Description of Predator Effects	References
* Delphastus catalinae* 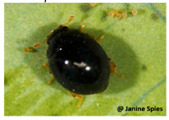	Consumption confirmed in laboratory Prefers eggs but feeds on other stages Presence increases dispersal of *B. tabaci* from cash crop Top greenhouse predator	[23,25,30,35,52]
*Collops vittatus* 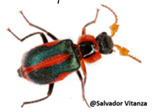	Consumption confirmed by MGCA ^2^ Negative effect on *B. tabaci* nymph and adult density in cotton fields Prefers eggs and adults Relatively abundant in southwest cotton fields	[17,19,36,38]
*Orius tristicolor* 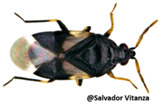	Consumption confirmed by MGCA Prefers adults but feeds on other stages >Can be very abundant in cotton fields	[18,19,29,36,38]
*Geocoris sp.* 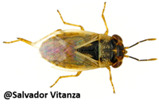	Consumption confirmed by MGCA Prefers adults and parasitized nymphs but feed on all life stages If present in high densities, insecticides may not be needed Can be very abundant in cotton fields	[19,29,36,38,43,46]
*Dapetis nr. divergens* 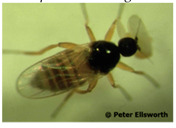	Consumption confirmed by MGCA Significant negative effect on *B. tabaci* nymphs and adult density in cotton fields Prefers adults but feed on other stages Can be very abundant in southwest cotton fields	[18,19,37]
*Misumenops celer* 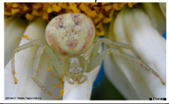	Consumption confirmed by MGCA If present in high densities, insecticides may not be needed Life stage preference unknown Can be abundant in cotton fields	[17,18,19,27,59]

^1^ The photo credit is as follows: (1) ©Janine Spies, (2–4) ©Salvador Vitanza, (5) ©Peter Ellsworth and (6) ©Joseph Berger, respectively, as ordered in predator column. ^2^ MGCA is the abbreviation for molecular gut content analysis.
